# Automated haematology analysis to diagnose malaria

**DOI:** 10.1186/1475-2875-9-346

**Published:** 2010-11-30

**Authors:** Germán Campuzano-Zuluaga, Thomas Hänscheid, Martin P Grobusch

**Affiliations:** 1 Grupo Malaria, Facultad de Medicina, Universidad de Antioquia, Calle 62 #52-59, Lab 610, Medellin, Colombia; 2 Laboratorio Clínico Hematológico, Carrera 43C No. 5-33, Medellín, Colombia; 3 Instituto de Microbiologia and Instituto de Medicina Molecular, Faculdade de Medicina, Av. Prof. Egas Moniz, 1649-028 Lisbon, Portugal; 4 Infectious Diseases, Tropical Medicine and AIDS, Division of Internal Medicine, Academic Medical Centre, University of Amsterdam, Meibergdreef 9, PO Box 22660, 1100 DE Amsterdam, The Netherlands; 5 Institute of Tropical Medicine, University of Tübingen, Germany; 6 Division of Infectious Diseases, Department of Internal Medicine, Faculty of Health Sciences, University of the Witwatersrand, Johannesburg, South Africa

## Abstract

For more than a decade, flow cytometry-based automated haematology analysers have been studied for malaria diagnosis. Although current haematology analysers are not specifically designed to detect malaria-related abnormalities, most studies have found sensitivities that comply with WHO malaria-diagnostic guidelines, i.e. ≥ 95% in samples with > 100 parasites/μl. Establishing a correct and early malaria diagnosis is a prerequisite for an adequate treatment and to minimizing adverse outcomes. Expert light microscopy remains the 'gold standard' for malaria diagnosis in most clinical settings. However, it requires an explicit request from clinicians and has variable accuracy. Malaria diagnosis with flow cytometry-based haematology analysers could become an important adjuvant diagnostic tool in the routine laboratory work-up of febrile patients in or returning from malaria-endemic regions. Haematology analysers so far studied for malaria diagnosis are the Cell-Dyn^®^, Coulter^® ^GEN**·**S and LH 750, and the Sysmex XE-2100^® ^analysers. For Cell-Dyn analysers, abnormal depolarization events mainly in the lobularity/granularity and other scatter-plots, and various reticulocyte abnormalities have shown overall sensitivities and specificities of 49% to 97% and 61% to 100%, respectively. For the Coulter analysers, a 'malaria factor' using the monocyte and lymphocyte size standard deviations obtained by impedance detection has shown overall sensitivities and specificities of 82% to 98% and 72% to 94%, respectively. For the XE-2100, abnormal patterns in the DIFF, WBC/BASO, and RET-EXT scatter-plots, and pseudoeosinophilia and other abnormal haematological variables have been described, and multivariate diagnostic models have been designed with overall sensitivities and specificities of 86% to 97% and 81% to 98%, respectively. The accuracy for malaria diagnosis may vary according to species, parasite load, immunity and clinical context where the method is applied. Future developments in new haematology analysers such as considerably simplified, robust and inexpensive devices for malaria detection fitted with an automatically generated alert could improve the detection capacity of these instruments and potentially expand their clinical utility in malaria diagnosis.

## Malaria diagnostic methods - 'where to use what'

For over a century microscopy has been the standard method for routine malaria diagnosis [1], allowing species identification and determination of parasitaemia, with a detection threshold of 4 to 100 parasites/μl [2]. Microscopy-based diagnosis is performed mostly in areas of low to moderate transmission, for example Latin-America, or parts of Asia and South Africa [3]. Interestingly, and despite the experience of microscopists, studies from endemic countries, such as India and South Africa, have shown that laboratory misdiagnosis is not uncommon [4,5]. This may be due to the immense workload and limited human resources. Laboratory misdiagnosis may also occur in developed countries with imported malaria [6], as laboratories in these areas deal with few cases annually, thus making it difficult to maintain the laboratory expertise in microscopic diagnosis. The need for well-trained microscopists, lack of equipment and/or periodic training, has led to the development of several alternative diagnostic methods [7]. Also, immunochromatographic rapid diagnostic tests (RDTs) have become widespread. In resource-poor areas, usually those with high malaria transmission rates, expensive artemisinin-based combination therapies are increasingly used, and this has led to the promotion of RDTs by malaria control programmes, as stipulated by WHO [8], as a prerequisite to 'informed' therapy with artemisinin combination therapy (ACT) [9].

Early parasitological malaria diagnosis is required to guide proper treatment and reduce adverse outcomes associated with the infection [10]. Lack of clinical and laboratory experience, prolonged incubation periods and *Plasmodium vivax *relapses [11,12], or prophylaxis in travellers [13] can delay diagnosis, thus increasing malaria associated adverse outcomes [14], especially in non-endemic countries. In a Canadian study , in patients with imported malaria, 45% infected with *P. vivax*, 33% with *Plasmodium falciparum*, 22% with other species or mixed malaria, 59% cases were missed on first presentation and 16% had ≥3 physician-contacts before malaria smears were ordered [15]. A study evaluating 185 malaria-related fatalities in travellers returning to the United States, of which 92.7% were caused by *P. falciparum*, 3.3% by *P. vivax *and the remainder by other species, found that 67.8% of these patients were not diagnosed in the first visit, 17.9% were diagnosed at autopsy, and 66.7% of preventable deaths were attributed to management failure upon presentation [14]. A reliable detection method for malaria incorporated into the routine complete blood count (CBC) could help detect cases earlier and potentially reduce adverse outcomes related to malaria infection.

## Discovery of automated haematology analysers for malaria diagnosis

The CBC is one of the most frequently requested laboratory tests in clinical medicine [16] with multiple indications, including the evaluation of febrile patients that could have malaria [17]. Furthermore, these instruments are available throughout the developed, and also increasingly so in the developing world. Most of these instruments are based on flow cytometry and have proven to be of value for malaria diagnosis [18,19]. Back in 1953, the first haematology analyser developed by Wallace Coulter was based solely on impedance detection (Coulter Principle) [16,20]. These instruments have evolved quickly and have incorporated chemical methods, direct current impedance, radiofrequency conductance, flow cytometry multiple-angle light scatter, and nucleic acid fluorescence detection methods for characterization of the blood cell populations, and provide highly accurate CBCs [21,22]. For those interested in the basic mechanisms by which haematology analysers measure and characterize blood corpuscles we refer the reader to several good reviews [21,23].

Until the early 1990s, anecdotal cases of malaria-related alterations in the CBC were described; for example, abnormal extra peaks in the white blood cell (WBC) histograms of a Coulter^® ^MaxM analyser (Beckman-Coulter, Inc, Miami, FL, USA) (Germán Campuzano-Maya, personal communication) and pseudoreticulocytosis in a Sysmex R-1000 (Sysmex Corporation, Kobe, Japan) [24]. In 1993, a study analysing 18 samples from patients with malaria (*P. falciparum: *10 and *P. vivax*: 8) and 52 samples from healthy controls with a Technicon H1^® ^analyser (Technicon Instruments Corporation, Tarry Town, NY; now Siemens), found that all malaria-infected patients had ≥3% (range 3.3-20.9%) of so-called 'large-unstained-cells' suggesting their potential use for malaria screening [25]. Unfortunately, changes in WBC populations [26], reticulocytosis, or increase of 'large-unstained-cells' [25] may also appear with other pathologies, giving these changes low accuracy for malaria detection.

The interest in haematology analysers was renewed after a first report showing that a Cell-Dyn^® ^(CD) analyser (Abbott Diagnostics, Santa Clara, CA, USA) allowed for a rather specific detection of malaria pigment in leukocytes [18]. This discovery led to a series of studies (Table 1 and Additional File 1), which have confirmed the potential of these instruments to aid in the diagnosis of malaria. In more recent years, other researchers have set out to investigate if other haematology analysers could also detect malaria, focusing on the Coulter^® ^GEN**·**S and LH 750 (Beckman Coulter, Inc, Miami, FL, USA), and the Sysmex XE-2100^® ^(Sysmex Corporation, Kobe, Japan). This review describes relevant key features of these analysers, characteristic malaria-related findings, diagnostic accuracy, clinical applications and limitations, as well as future directions of this novel malaria diagnosis modality.

**Table 1:** Summary of studies evaluating the malaria diagnostic accuracy of Cell-Dyn series analysers using the side-scatter/depolarized side-scatter plot abnormal depolarizing events criterion. §

First author, year and country	Number of participants and diagnoses	**Index test criterion**^**¶**^	Sensitivity %	Specificity %
Mendelow, 1999, South Africa [18]	Total: 224 directed samples from 175 patients, *P. falciparum: *93, Species not specified: 2	CD** 3500 ≥1 depolarizing events‡	72	96
Hänscheid, 2001, Portugal† [39]	Total: 174, *P. falciparum*: 48, *P. vivax*: 6, *P. ovale*: 1, *P. malariae*: 2	CD 3500 ≥2 depolarizing events	95	88
Wever, 2002, The Netherlands† [36]	Total: 113, *P. falciparum*: 46, *P. vivax*: 5, *P. ovale*: 4, no differentiation for *P. vivax *or *P. ovale*: 3	CD 3500 Either ≥1 depolarizing events or pseudoreticulocytosis	62	96
Grobusch, 2003, Germany† [27]	Total: 403, *P. falciparum*: 87, *P. vivax*: 13, *P. ovale*: 5, *P. malariae*: 2	CD 3000 ≥1 depolarizing events	48.6	96.2
Scott, 2003, South Africa [35]	Total: 831, *P. falciparum*: 334, *P. vivax*: 7, *P. ovale*: 1, *P. malariae*: 2, mixed or unspecified: 6	CD 4000 ≥1 depolarizing events	80.2	87.3
Suh, 2003, South Korea [32]	Total: 168, *P. vivax*: 68	CD 4000 ≥1 depolarizing events	91.2	100
Dromigny, 2005, Senegal [30]	Total: 799 (directed: suspected of malaria 123, non-suspected random samples 676) *P. falciparum*: 68, treated or subclinical: 83	CD 3200 ≥1 depolarizing events	Directed 92.9 Random 90.2	Directed 93.8 Random 96.7
Padial, 2005, Equatorial Guinea [41]	Total: 411, *P. falciparum*: 35 , *P. ovale*: 3, mixed: 1	CD 4000	72	98
Josephine, 2005, Malaysia [40]	Total: 889, *P. vivax*: 12, *P. malariae*: 3, *P. falciparum*: 1	CD 4000	100	100
de Langen, 2006, Namibia [42]	Total: 208, *P. falciparum*: 90	CD 3700 ≥1 depolarizing events	93	97
Hänscheid, 2008, Gabon [34]	Children, total: 368, *P. falciparum*: 152	CD 3000*** ≥1 depolarizing purple events Green-coded events	a) 96% b) 85%	a) 96% b) 96%
Hänscheid, 2009, Gabon [43]	Pregnant patients, total 685, *P. falciparum*: 86	CD 3000 ≥1 depolarizing events	86.8	78.5
Rathod, 2009, India [33]	Total: 523, *P. falciparum *:73, *P. vivax*: 62	CD 3200 ≥1 depolarizing events	62.2	25.3

^**¶**^Index diagnostic test: abnormal depolarizing events in the side-scatter/depolarized side-scatter plot. ‡All studies use the instrument's diagonal separation line for eosinophils and neutrophils in the side-scatter/depolarized side-scatter plot, unless otherwise specified. ** CD: Cell-Dyn. †Imported malaria. ***For purple events, these were considered positive if present above a line traced at 5 pixels from the *x *axis. For green events, a special gate was created to identify haemozoin-laden granulocytes, with the intention to exclude eosinophils. In accordance with studies using flow cytometric cell sorting [27], the largest possible gate to the left and above the usual location of the eosinophil population was created which did not contain any eosinophils [34]. For this, CBC analyses from children without malaria or pseudoreticulocytosis were used [34]. ^**§**^For the complete table with additional comments and reference diagnostic tests used in each study see Additional File 1.

## Cell-Dyn analysers and detection of malaria pigment (haemozoin)

**Figure 1:**
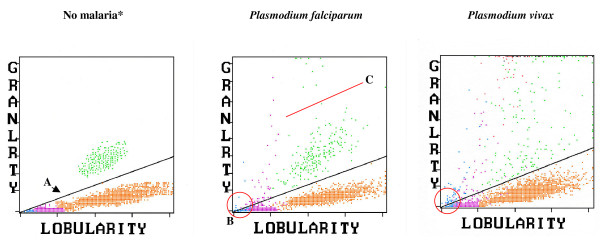
**Cell-Dyn 3700 side-scatter/depolarized side-scatter plot of samples with no malaria, *P. falciparum *and *P. vivax *malaria**. **A**. The diagonal line gives optimal separation between eosinophils (green) and neutrophils (orange). In the *P. falciparum *sample (middle panel) purple dots indicate depolarizing monocytes. At the top of the scatter-plot are haemozoin-containing neutrophils misclassified as eosinophils. Blue-coded depolarizing events might possibly be small haemozoin-containing monocytes. In the *P. vivax *sample (right panel), the changes are more pronounced, and additionally haemozoin-containing RBC (red) appear to be present. **B**. As the diagonal line reaches the 0/0 point (red circle), small increases in depolarization may cause monocyte/lymphocyte events to easily surpass this line and be classified as depolarizing (false positives). **C**. In the middle panel it is easy to distinguish two green-coded populations below (eosinophils) and above the red line (haemozoin-containing neutrophils), which is not always the case (see *P. vivax *sample). * Colour code for events displayed is, blue: lymphocytes; purple: monocytes; orange: neutrophils; green: eosinophils; red: erythrocytes; black: not classified.

**Figure 2:**
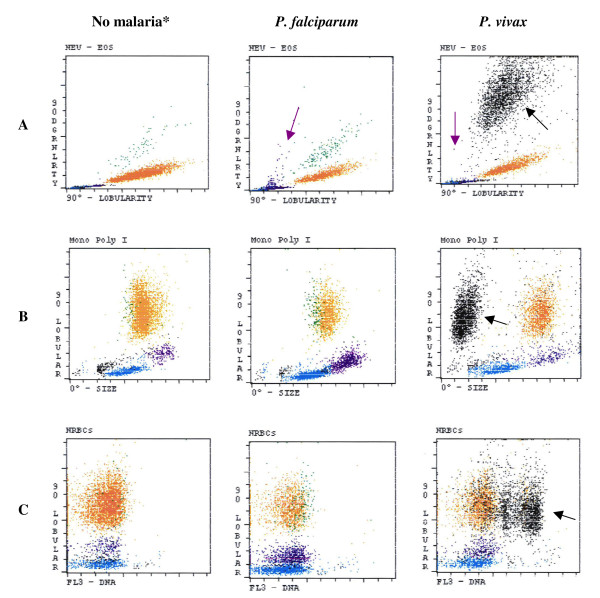
**Cell-Dyn 4000 scatter-plots of samples with no malaria parasites, *P. falciparum *and *P. vivax***. **A**. Haemozoin detection by depolarization in the NEU-EOS scatter-plot: Haemozoin-containing monocytes (purple dots) in eosinophil area (purple arrows). Large black-coded population (black arrow) in *P. vivax *infection (right scatter-plot). **B**. Detection by size and depolarized side-scatter in the 'mono poly I' scatter-plot: No difference between 'no malaria' and *P. falciparum*, while a large population of small size black-coded events appears in the sample with *P. vivax*. **C**. Detection of parasite DNA in the nucleated red blood cells (NRBC) scatter-plot by propidium iodide staining: No difference between 'no malaria' and *P. falciparum *while a large population of black-coded events with high degree of FL3 fluorescence appears in the *P. vivax *case. Black-coded events may represent parasites (see text), 90 Dgrnlrty: 90° depolarization side-scatter; 90° lobular: 90° side-scatter; 0° Size: forward-scatter; FL3-DNA: fluorescent detection of propidium iodide. *Colour code for events displayed is, blue: lymphocytes; purple: monocytes; orange: neutrophils; green: eosinophils; red: erythrocytes; black: not classified.

The Cell-Dyn instruments use laser light scatter at various angles, the so called multiple-angle polarized scatter separation for WBC analysis. Multiple-angle polarized scatter separation is used to distinguish eosinophils from neutrophils based on the light depolarizing properties of their granules, but has also been found to detect haemozoin-containing monocytes and granulocytes (Figures 1 and 2) [18,27-29]. These malaria-related events are shown in a scatter-plot with 90° side-scatter on the *x*-axis and 90° depolarized side-scatter on the *y*-axis, usually labelled as lobularity/granularity scatter-plot in the CD 3000 series or NEU-EOS in the CD 4000 (Figures 1 and 2).

Using a MoFlo^® ^high speed cell sorter (Beckman Coulter, Inc, Fullerton, CA), it was confirmed that the observed changes were caused by haemozoin-containing monocytes and neutrophils [27,29], as did a study where samples from monocytes, which had previously ingested haemozoin *in-vitro*, produced depolarizing events in the side-scatter/depolarized side-scatter plot of a CD 3200 [30]. Interestingly, there is evidence that depolarizing material in intra-erythrocytic parasites could also be detected [31,32]. It appears that excitation light with a longer wavelength causes slightly stronger depolarization signals [29]. Of note, the CD 3000 series uses a 633 nm red laser, while the CD 4000 and CD Sapphire use a 488 nm blue laser for excitation. However, it is still unclear if this difference has any impact on the sensitivity for malaria detection.

### Cell-Dyn analysers - colour code for events and cells detected

#### Purple dots. Depolarizing monocytes

The appearance of purple-coded events above the dynamic diagonal threshold line (Figure 1) separating eosinophils from granulocytes in the side-scatter/depolarized side-scatter plot is the most studied malaria diagnosis criterion for the Cell-Dyn analysers (Table 1). However, this default diagonal line intersects at the 0/0 value of the *x*/*y*-axis, potentially causing a progressive decrease in specificity as the *x *and *y *axes intersect when used as cut-off for malaria diagnosis. Depolarizing events that appear near this point (red circle in Figure 1), although showing low lobularity and granularity values (i.e. normal monocytes and lymphocytes) may still be present just above the dividing line, causing false positive results. Perhaps, this could explain an unusually low specificity of 25% reported in one recent study [33]. In fact, this finding has led some authors to construct horizontal lines for analysis [34], or an initial horizontal portion for the diagonal line (Figure 1); for example, only considering events above channel 25 on the *y*-axis [35,36], or even above channel 50 [30].

#### Green dots. Granulocytes miss-classified as eosinophils

Highly depolarizing green-coded events could be indicative of haemozoin-containing granulocytes since it is unlikely that these events are just the result of eosinophil granules [34,37]. Applying the Cell-Dyn set-up to a Mo-Flow high speed sorter allowed to show that green-coded events, which fall outside a given area, are likely haemozoin-containing granulocytes [29]. However, it is difficult to establish a clear cut-off between eosinophils and other granulocytes with less haemozoin, which depolarize less (Figure 1C). These haemozoin-containing granulocytes might project into the eosinophil population and thus may not be singled-out. However, in one study the eosinophil-free region was optimized for malaria detection by using malaria negative samples, and when considering any green dot in this area as positive, the sensitivity and specificity were 85% and 96%, respectively [34].

#### Blue dots. Abnormal lymphocytes or small monocytes?

Interestingly, in many cases blue-coded events, representing lymphocytes, show depolarization (Figure 1). In one study, this was observed in 77 of 152 children with malaria [34]. Yet, lymphocytes are not phagocytic cells. Although no study has looked systematically into this, it seems likely that the recent discovery of different monocyte subsets may offer an explanation [38], as some monocyte subpopulations may be rather small and show a round nucleus, similar to lymphocytes. The Cell-Dyn may thus miss-classify these cells as lymphocytes. If this is confirmed, depolarizing blue-coded events ought to be included as part of the side-scatter/depolarized side-scatter plot diagnostic criterion for malaria.

#### Red dots and black dots. Haemozoin-containing parasites?

One report using a CD 3700 has suggested that in blood with *P. vivax *parasites and osmotically-resistant RBCs, haemozoin inside infected erythrocytes may also be detected [31]. With the CD 3000, these events are correctly identified as RBC (red dots) (Figure 1). Using the CD 4000, in cases of *P. vivax *infections, large black-coded populations have been observed, which usually indicate non-leucocytic events (Figure 2). These events could be caused by parasites since these populations are much smaller in size than any of the leukocyte populations [32,35,37]. However, it is unclear if they represent lysis-resistant haemozoin-containing erythrocytes or perhaps large parasites seen in mature forms (mature trophozoites, schizonts). The suspected nature of these events could explain why this pattern is more frequent in *P. vivax *infections, which have asynchronous parasitaemia and more circulating mature parasites rich in haemozoin [37].

### Cell-Dyn analysers and haemozoin-containing WBCs - studies on diagnostic accuracy

The most-studied malaria-related abnormality for Cell-Dyn analysers is the presence of abnormal monocyte-coded depolarizing events (purple) in the side-scatter/depolarized side-scatter plot (Table 1 and Additional File 1). The first published study, by Mendelow and colleagues in South Africa (1999), tested 224 blood samples from patients with suspected malaria (99% with *P. falciparum*) and found that ≥1 purple-coded event(s) above the diagonal eosinophil/granulocyte threshold line (Figure 1) had a sensitivity of 72% and a specificity of 96% [18]. However, the definition of what constitutes a Cell-Dyn malaria-positive sample varies between studies, especially regarding the position of the cut-off line and the fact that multiple reference diagnosis tests are used (Table 1). Sensitivity in 13 studies ranged from 48.6% to 100% [18,27,30,32-37,39-43] and specificities from 25.3% to 100% [18,27,30,32-37,39-43] (Table 1). Some studies on imported malaria showed rather low values for sensitivity (Table 1); for example 48.6% found in Germany [27], 55% found in South Africa [44], and 62% in The Netherlands [36]. Interestingly, Mendelow and colleagues showed that sensitivity was 90% in black patients *versus *43% in white patients [18]. The best explanation for this is that non-immune patients, more frequently white, might have very few circulating haemozoin-containing leukocytes when surpassing the pyrogenic threshold, i.e., at the time when malaria symptoms occur [27]. In line with this, the sensitivity in a study from Portugal was 95% [39], with malaria occurring almost exclusively in black African immigrants from former colonies, with the possibility of residual immunity levels. Many presented rather late during their malaria episode, when the haemozoin burden would be expected to be higher. Grobusch and colleagues found that the sensitivity of the CD 3000 to detect malaria in semi-immune patients, as measured by indirect fluorescent malaria antibody test (titre ≥1:40), was 73.7% compared to 28.6% in non-immune patients [27]. This discrepancy was related to the concentration of haemozoin-laden macrophages in each group, with semi-immune and non-immune patients having a median relative frequency of 9 × 10^-4 ^and 1.5 × 10^-4 ^cells respectively[27]. Consequently, in most malaria-endemic countries, sensitivity values of >90% were not uncommon (Table 1).

Contrary to sensitivity, values for specificity regularly reached 90% across studies, with many studies reporting figures close to 100% (Table 1). However, one outlier study reported a highly discrepant value of only 25% specificity, due to technical limitations when using the built-in diagonal separation line, as almost all false positives (269/290) showed only a single depolarizing purple dot [33]. As explained above, these events would have counted as non-significant in most settings and this shortcoming can be corrected by using an initial horizontal portion for the separating line (Figure 1) [30,35,36].

### Cell-Dyn analysers - further malaria-related changes

Other malaria related abnormalities described only for the CD 4000 include abnormalities observed in other scatter-plots, especially the EOS-I scatter-plot, which shows granularity (depolarized side-scatter) *versus *size (0°-forward-scatter) [32,35,37,41]. Several studies reported the appearance of highly granular (depolarized side-scatter values above channel 50), green or black-coded events clustered (as opposed to random) in the small-size range (low forward-scatter) [32,35,37]. Given the small size and high granularity of these events, they most likely correspond to haemozoin-laden mature parasites or haemozoin-rich red cell 'ghosts' [37]. Events in the depolarized side-scatter/forward-scatter plot (EOS-I, CD 4000) are more frequent with *P. vivax *[35,37]. Clustered events on the EOS-I scatter-plot have sensitivities that range from 57.1% to 93% for *P. vivax *but are as low as 0% to 1.5% for *P. falciparum*, and are near 100% specific for both *Plasmodium *species [35,37]. Similar events can also be observed in the mono-poly I scatter-plot (side-scatter/forward-scatter plot) (Figure 2) [45]. Also, propidium iodide, used in the CD 4000 to detect nucleated RBC stains parasites, thus causing abnormal signals in the scatter-plots that register the FL3 signal (red fluorescence) (Figure 2) [37,45]. Interestingly, these events may also be more frequent in *P. vivax *infections [37,45].

Finally, reticulocyte-associated changes have been reported, usually in *P. falciparum *samples [36,44], such as extra spikes in the reticulocyte histogram, pseudoreticulocytosis, and a high immature reticulocyte fraction [41,44,46]. One study evaluated 108 *P. falciparum*-infected samples and found a good correlation (*R*^*2 *^= 0.6) between parasitaemia and Cell-Dyn reticulocyte percentages [44], and all samples with a parasitaemia superior to 5% infected RBCs had an immature reticulocyte fraction ≥0.5; of those with an immature reticulocyte fraction ≥0.5, 81% had an isolated spike on the reticulocyte histogram [44]. However, due to expensive reagents, hardly any laboratory runs the haematology analyser in the reticulocyte mode on a routine basis, decreasing the utility of any malaria-related finding in the reticulocyte measurements.

### Cell-Dyn analysers - changes observed in P. vivax and other species

The frequency and patterns of malaria-related events in the CD 4000 scatter-plots differ between *Plasmodium *species (Figure 2) [32,35,37,45]. As described above, small sized black-coded populations with high degree of depolarization were noted with increased frequency in *P. vivax*-containing samples. In one study using a CD 4000, 12 out of 20 *P. vivax*-positive samples consistently showed depolarizing events after being depleted of WBC, suggesting that in *P. vivax *malaria these events may also be directly caused by parasites [32]. The same authors analysed serially diluted leukocyte depleted samples with a CD 4000 and determined a detection threshold for depolarizing events of 288 ± 17.7 parasites/μl [32]. Another study also showed small clustered black-coded events in the EOS-I scatter-plot (depolarized side-scatter/forward-scatter) being seen almost exclusively in *P. vivax *cases [37]. Given the small size and high depolarization values for these events, they were most likely caused by haemozoin-laden mature parasites or haemozoin-rich red cell 'ghosts' [37], coinciding also with the asynchronous parasitaemia of *P. vivax*. *Plasmodium ovale *and *Plasmodium malariae *infections have shown abnormal side-scatter/depolarized side-scatter plots [36,40].

### Cell-Dyn analysers and malaria detection - limitations and necessary improvements

Haemozoin ingested by neutrophils may be detected for up to a median of 72 hours and haemozoin ingested by monocytes up to a median of 216 hours according to each cell's circulating half life [47]. Although this could translate into false positives in areas of high transmission, as patients with recent episodes of malaria may still harbour these cells, studies from endemic areas have not reported low specificities (Table 1). Dromigny and colleagues found that the false positive rate among convalescent patients who could still have circulating haemozoin-laden phagocytes was 17.6%; however, including these samples into the 'malaria negative' group only decreased specificity from 96.7% to 95.6% [30].

The detection threshold for malaria probably depends on total parasite burden and may vary with the Cell-Dyn model used. One study reported a reduction in sensitivity from 72% down to 67% with parasitemias in the range of 0.1% - 1%, and further down to 50% with parasitaemias of less than 0.1% [41]. The total number of haemozoin-containing cells in the body and those analysed and represented by the instrument determine the sensitivity [29,34]. Using the Mo-Flow cell sorter and rare event analysis, the threshold for detection of malaria by the Cell-Dyn instruments was in the order of 2 × 10^-4 ^pigment-containing monocytes [29]. However, most of the Cell-Dyn 3000 instruments only analyse WBC in a given volume, with an upper limit of 10^4 ^WBC. Not surprisingly, one study showed that only a mean of 1,364 monocytes (range: 230 to 3,660) were analysed in each sample (n = 152), indicating an intrinsic limitation of detection of these instruments [34]. Furthermore and more importantly, not all analysed cells are shown on-screen, and screen resolution is inferior for the CD 3000 series where 140 × 140 pixels represent results from 256 × 256 channels (around 3.34 times less information resolution) [34].

Additionally, only the first 5,000 events analysed are used for the graphic display, and in the case of cells being superimposed, only one event is shown [34]. Results from a study in Gabon that used screenshots indicate that each side-scatter/depolarized side-scatter plot of the CD 3000 series contains only around 500 pixels, representing the analysed cells, with a data-loss, based on manual on-screen analysis, that may be as high as 90% (Table 2) [34].

**Table 2:** Cell counts and data loss with a Cell-Dyn 3000 instrument.

Cell type	Mean cell count in CBC result	Mean number of cells analysed	Mean number pixels on screen*	Information (cell count) lost in scatter-plot display (%)**
WBCs	8675/μL	9100	482	94.4
Granulocytes	3834/μL	4174	348	90.9
Lymphocytes	2992/μL	3068	22	99.3
Monocytes	1334/μL	1379	58	95.7
Eosinophils	349/μL	345	43	87.7

Data shown correspond to the mean values for 153 CBC results from malaria positive patients, obtained with a CD 3000 instrument in Lambaréné, Gabon. *Coloured pixels indicate the events that represent analysed cells in the side-scatter/depolarized side-scatter plot (lobularity/granularity) on-screen (Figure 1). ** % data lost = 1- (Mean number pixels on screen/Mean cell count in CBC result) × 100. Screen images were analysed by taking a screenshot and counting the pixels using ImageJ image processing software.

In summary, the CD instruments appear to detect malaria associated changes with a high degree of reliability, however, this depends so far on the meticulous observation of the described changes during validation of CBC results (either on screen or print-out). Thus, laboratory staff ought to receive continuous training allowing them to recognize these changes. Obviously, it would be highly desirable if the manufacturer included analysis algorithms that would automatically flag suspicious samples.

## Coulter GEN·S and LH analysers

**Figure 3:**
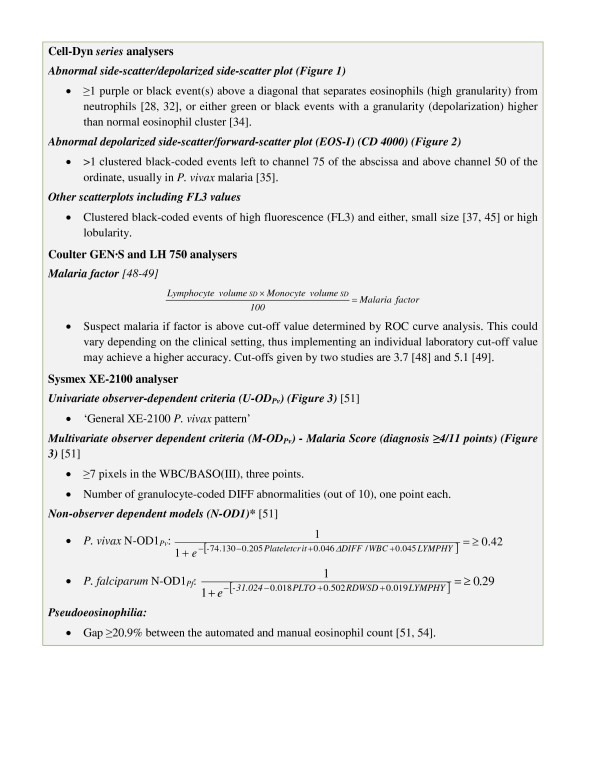
**Summary of proposed malaria diagnostic criteria for the Abbott Cell-Dyn, Coulter GEN·S and LH 750, and Sysmex XE-2100 haematology analysers**. *Non-Observer Dependent (N-OD) models use the logistic regression predicted probability equation: $\frac{1}{1+e^{-\left(\beta_{0}+\beta_{1}x_{1}+\beta_{2}x_{2}+...+\beta_{n}x_{n}\right)}}=\text{PP}$, where *β*_*0 *_and *β*_*1,2, ... n *_correspond to the intercept and variable's coefficients, *x*_*1,2, ... n *_are the values for each variable obtained for each individual blood sample, and PP is the predicted probability for which the optimal diagnosis cut-off is show in the figure [51]. Samples with a 'Predicted Probability' (PP) above the cut-off are considered positive for malaria and could be flagged by a programmed Laboratory Information System. Variables for N-OD1_*Pv*_: plateletcrit; ratio between DIFF channel and total WBC count (ΔDIFF/WBC); and mean value of LYMPH-Y in arbitrary units (LYMPHY). Variables for N-OD1_*Pf*_: Optical platelet count (PLTO); red cell distribution width SD (RDWSD); and LYMPH-Y in arbitrary units (LYMPHY).

Coulter GEN·S and LH 750 haematology analysers use Volume-Conductance-Scatter (VCS) technology to obtain 'positional parameters' of all WBC by measuring impedance for cell volume; radiofrequency conductivity for internal structure and nuclear characteristics; and flow cytometry-based helium-neon laser light scatter analysis for cellular granularity, nuclear lobularity and cell surface structure [22,48]. An initial study by Fourcade and colleagues (2004), using a GEN**·**S that included 89 participants suspected of having malaria (28 had *P. falciparum *and four had *P. vivax*) compared all positional parameters between malaria-negative and positive samples. They determined that the lymphocyte volume standard deviation (SD) and the monocyte volume SD were the most accurate for malaria detection [49]. These abnormalities reflect cellular anisocytosis, probably from activated monocytes as a response to the infection [48]. Fourcade and colleagues proposed a discriminant factor ('malaria factor' [48]) using the lymphocyte SD and monocyte SD values (Figure 3)[49]. Receiver operator curve (ROC) analysis determined an optimal cut-off value of 5.1 for a sensitivity and specificity of 96.9% and 82.5%, respectively [49]. In a subsequent study with 275 participants suspected of malaria (147 with malaria), 1079 healthy volunteers and 51 HIV infected patients without malaria, Briggs and colleagues calculated a malaria factor of 3.7 with a sensitivity and specificity of 98% and 94%, respectively (Table 3) [48]. The malaria factor for *P. falciparum, P. vivax*, and *P. ovale *was 6.2, 5.9 and 5.7, respectively [48]. In both studies, some malaria-positive samples showed an extra peak in the WBC histogram at the 35 fl threshold [48,49]. Briggs and colleagues determined that the malaria factor, in the absence of an extra peak in the WBC histogram, a platelet count ≥150 × 10^9^/l, eosinophils >0.015%, monocyte volume SD <23.2 fl and a mean volume for monocytes <180 fl had a negative predictive value of 99.7% [48]. Samples with a positive malaria factor had a parasitaemia ranging from 0.001% to 38.9% infected RBCs [48]. By incorporating of the calculation for the malaria factor into the Coulter analyser's Information Processing Unit or into the Laboratory Information System an automated malaria alarm for this type of analyser could be implemented.

**Table 3:** Summary of studies evaluating malaria diagnostic accuracy of the Coulter GEN·S and LH 750 analysers.

First author, year and country	Number of participants and diagnoses	Standard reference test	Blinding	Malaria factor	Sensitivity %	Specificity %
Fourcade, 2004, France and Spain [49]	Total: 89, *P. falciparum*: 28, *P. vivax*: 4	Microscopy, HRP2+ pan-malarial antigen (Binax Now)	-	5.1	82.5	96.9
Briggs, 2006, South Africa and England [48]	Total: 1354, healthy: 1079, febrile: 135, HIV infected: 51, *P. falciparum*: 120, *P. vivax*: 11, *P. ovale*: 7, *P. malariae*: 1, mixed *P. falciparum *and *P. vivax*: 1	Microscopy, QBC, HRP2+ pan-malarial antigen (Binax Now), *Pf*HRP2 (MAKROmed), *P*-LDH (optiMAL)	-	3.7	98	94
Kang, 2008, South Korea [66]*	Total: 395, *P. vivax*: 68	Microscopy	**	4.57	81.8	72.3

*Article in Korean, only abstract in English. **Could not be assessed.

## Sysmex XE-2100 analyser

**Figure 4:**
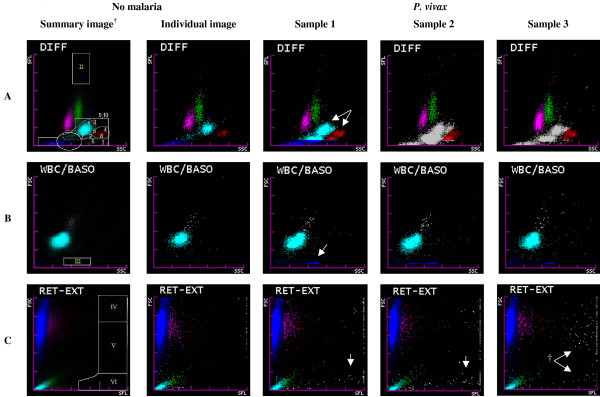
**Normal and abnormal Sysmex XE-2100 scatter-plots where *P. vivax*-related findings have been identified**. ^†^Sysmex XE-2100 summary images composed of 50 superimposed images from samples without malaria, with lines delimiting where *P. vivax-*associated abnormalities appear. 1. neutrophils, outside limit (yellow line); 2. neutrophils, inferior deviation; 3. neutrophils, right deviation; 4. eosinophils, outside limit (yellow line); 5. confluent neutrophils and eosinophils; 6. granulocytes outside inferior limit; 7. ≥2 neutrophil-coded groups; 8. ≥2 eosinophil-coded groups; 9. tendency of granulocytes to form one group; 10. abnormal granulocyte colour (gray or normal). Variables 1 to 10 are used to obtain the Malaria Score (M-OD_*Pv*__, _Figure 3). Pixel-counting areas were malaria related events appear are DIFF(I), DIFF(II), WBC/BASO(III), RET-EXT(IV), RET-EXT(V) and RET-EXT(VI). **A**. DIFF (SSC *versus *SFL) scatter-plot shows lymphocytes (magenta), monocytes (green), neutrophils (sky blue), eosinophils (red) and RBC ghosts (blue), non-identified events (gray). The malaria related abnormalities are shown in the images from three samples with '*P. vivax*', for example, the duplication and fusion of the neutrophil and eosinophil groups (arrows) and gray-coded groups. **B**. WBC/BASO (SSC *versus *FSC) scatter-plot: Separates WBCs (sky blue) from basophils (gray). Malaria-related findings can be seen WBC/BASO(III) counting area (arrow). Malaria related findings that appear in the high SSC range of both the DIFF and WBC/BASO scatter-plot could be caused by haemozoin crystals in mature parasites [51]. **C**. RET-EXT (SFL *versus *FSC) scatter-plot: FSC differentiates RBC (high FSC, blue-magenta-red SLF progression) and platelets (low FSC, sky blue-green SFL progression) based on their size. Gray events usually correspond to WBC nuclear debris (high SFL). However, in *P. vivax *infected samples, †gray-coded events with middle and low FSC (arrows) and high SFL values can be found in the RET-EXT(V) and (VI) counting areas and could be generated by the parasite's nucleic acids [51].

The Sysmex XE-2100 automated haematology analyser uses combined impedance and radiofrequency conductance detection, semiconductor diode laser light 90° side-scatter (SSC) and 0° frontal-scatter (FSC) detection, and polymethyne fluorescence nucleic acid staining 90° side-fluorescence (SFL) detection. It measures 32 clinical variables, and graphs seven scatter-plots and two histograms [22,50]. Three scatter-plots have shown significant abnormalities in samples with malaria (Figure 4) [51].

### Abnormal DIFF, WBC/BASO and RET-EXT scatter-plots, and pseudoeosinophilia

In South Korea, two case series, one with sixteen [52] and the other with three [53]* P. vivax*-infected patients, reported spuriously elevated eosinophil counts (pseudoeosinophilia) and abnormalities in the DIFF scatter-plot consisting of additional blue, red or gray-coded grouped events, and a fusion of both neutrophil and eosinophil groups (Figure 4) [52,53]. Later, two studies in a malaria-endemic region in South Korea evaluated pseudoeosinophilia (>5% difference between the automated and manual eosinophil count) and DIFF scatter-plot abnormalities for *P. vivax *diagnosis against thick film [54], or against thick film and real-time polymerase chain reaction (RT-PCR) [55] (Table 4). In the first study by Huh and colleagues [54], pseudoeosinophilia and abnormal DIFF scatter-plot alone yielded sensitivities of 39% and 52%, respectively, with no change in specificity. In the more recent study by Yoo and colleagues [55], the positive and negative predictive values were 97.9% and 86.2%, respectively, and an abnormal DIFF scatter-plot alone yielded a marginal sensitivity of 16%. This large decrease in sensitivity for DIFF abnormalities could arise from the lack of a consensus definition for this diagnosis criterion, as well as difficulty with, and subjectivity in manually evaluating these patterns that could have resulted in a classification bias towards highly abnormal patterns in the latter study [55]. Regarding pseudoeosinophilia, a more recent study suggests a higher optimal cut-off of 21%, for *P. vivax*, with clinically insufficient accuracy (57.5%) [51]. Furthermore, pseudoeosinophilia warrants little clinical usefulness, since the microscopy required for its calculation would equally enable the visualization of parasites. It is also noteworthy that abnormalities in the DIFF scatter-plot and WBC counts of the XE-2100 have also been reported in samples with *Candida *spp. [26].

**Table 4:** Summary of studies evaluating the malaria diagnostic accuracy of the Sysmex XE-2100 analyser.

First author, year and country	Number of participants and diagnoses	Blinding	Index test criterion		Sensitivity %	Specificity %
Huh, 2008, South Korea [54]	Total: 463, *P. vivax*: 144	-	>5% pseudoeosinophilia and/or an abnormal DIFF scatter-plot^†^		69.4	100
Yoo, 2010, South Korea [55]*	Total: 1801, *P. vivax*: 413	-			46.2	99.7
Campuzano-Zuluaga, 2010, Colombia [51]	Total: 158, *P. falciparum*: 30, *P. vivax*: 65, 63 febrile patients Validation control group including healthy and febrile participants: 161	+	**Model**^**¶**^	**Variables (Figure 4)**		
			U-OD_*Pv*_^‡^	'XE-2100 *P. vivax *pattern'	96.9	93.6
			M-OD_Pv_**	Number of granulocyte DIFF abnormalities ≥7 pixels in the WBC/BASO(III)	95.4	98.4
			N-OD1_*Pv*_	ΔDIFF/WBC Plateletcrit LYMPH-Y	94.3	95.1
			N-OD2_*Pv*_	PLT-O Pixels WBC/BASO(III)	96.8	96.8
			N-OD1_*Pf*_	PLT-O RDW-SD LYMPH-Y	93	81
			N-OD2_*Pf*_	PLT-O RDW-SD Pixels WBC/BASO(III)	86	90

* All studies use microscopy as reference test for malaria diagnosis, except Yoo and colleagues study that also use real time quantitative PCR (RT-PCR). ^†^An abnormal DIFF scatter-plot corresponds to extra blue, red or gray-coded groups in the DIFF scatter-plot (Figure 4). ^¶ ^All N-OD models use continuous variables and are designed to compute a predicted probability of malaria obtained from the logistic regression analyses as shown in Figure 3. ^‡^The recognition of a 'general XE-2100 *P. vivax *pattern' seen in the DIFF, WBC/BASO and RET-EXT scatter-plots corresponds to the univariate observer-dependent model [U-OD_*Pv*_]. M-OD_*Pv*_: The multivariate observer-depend model, by means of a 'malaria score' quantifies several abnormalities seen in the U-OD_*Pv*_. **P. vivax Malaria Score: '≥7 pixels in the WBC/BASO(III)' (3 points), 'number of granulocyte-coded DIFF abnormalities' (10 variables; 1 point per variable), with ≥4 points being diagnostic. N-OD1: Non-observer dependent models that use built-in XE-2100 variables, for *P. vivax *(_*Pv*_) and *P. falciparum *(_*Pf*_). N-OD2: Non-observer dependent models that use built-in XE-2100 variables and the WBC/BASO(III) pixel count (Figure 4), for *P. vivax *(_*Pv*_) and *P. falciparum *(_*Pf*_). PLT-O: Optic platelet count. RDW-SD: Red cell distribution width SD.

In a recent study in Colombia, Campuzano-Zuluaga and colleagues found that *P. vivax *samples also showed salient abnormalities in the WBC/BASO and RET-EXT scatter-plots (Figure 4) [51]. For the WBC/BASO(III) counting area, ≥7 blue-coded events (pixels) had a sensitivity and specificity of 97% and 94%, respectively, for *P. vivax*; and ≥3 pixels had a sensitivity and specificity of 60% and 67%, respectively, for *P. falciparum*. For the RET-EXT(V and VI) counting areas, ≥1 linear or clustered gray events (pixels) had a sensitivity and specificity of 77% and 76%, respectively, for *P. vivax *(Figure 4) [51]. This study found a significant moderate-to-high correlation for most abnormalities in the DIFF, WBC/BASO and RET-EXT scatter-plots and the concentration of *P. vivax *mature trophozoites, schizonts or gametocytes (Figure 4) [51]. However, studies tailored to establish the nature of these abnormalities are needed, for example by measuring leukocyte-reduced samples with synchronized parasitaemia.

### An algorithmic approach to malaria detection with the XE-2100

The numerous XE-2100 variables affected by malaria parasites, up to 35 variables in *P. vivax *infections, make their individual use for malaria diagnosis cumbersome and unpractical [51]. However, by mathematically combining variables with the highest accuracy into a single diagnostic criterion, the method can be optimized and simplified into a robust numerical algorithm, computable by a Laboratory Information System or even by the Sysmex Information Processing Unit in the future. With this premise, Campuzano-Zuluaga and colleagues carried out a study where diagnostic models for both *P. vivax *(n = 65) and *P. falciparum *(n = 30) where developed and evaluated for accuracy against 63 samples from febrile patients, and partially validated against samples from a composite group of 161 febrile patients and healthy individuals (Table 4). They selected the best malaria-predictor variables and in a step-wise approach created several multivariate logistic regression prediction models. The optimal diagnostic 'predicted probability' (PP, numerical output of the model) as determined by ROC curve analysis was used as the diagnosis criterion for each model (positive: ≥ optimal PP; negative: < optimal PP) (Figure 3 and Table 4) [51]. Besides creating two observer-dependent models for *P. vivax *diagnosis, which require trained personnel to evaluate them manually, the authors developed two non-observer dependent (N-OD) models for each species. One used XE-2100 built-in variables (N-OD1), and the other one used built-in variables and new scatter-plot variables defined by the authors (N-OD2) (Figure 3, Figure 4 and Table 4) [51]. These models could allow for the systematic screening and detection of samples with malaria in a case-by-case fashion without the intervention of laboratory personnel in the procedure, and their accuracies make them promising for clinical application and testing in population-based studies (Table 4).

The N-OD1_*Pv *_and N-OD1_*Pf *_models include the increase in the mean number of events detected in the LYMPH-Y (*y *axis) channel [51], which has shown a moderate association with ring forms of *P. vivax *(*R*^*2 *^= 0.206, *P *= 0.01) and *P. falciparum *(*R*^*2 *^= 0.305, *P *= 0.03) parasites [51]. The ΔDIFF/WBC variable in the N-OD1_*Pv *_corresponds to the ratio between the WBC counts in the DIFF channel over the total WBC count multiplied by 10^3 ^and may relate to an excess count of malaria related events in the DIFF channel [51]. The N-OD2 model for both species include the WBC/BASO(III) pixel count which was found to be the best malaria-diagnostic variable alone and in combination with other variables (e.g. thrombocytopenia) (Table 4 and Figure 4) [51]. Events in the WBC/BASO(III) are not currently quantified by the XE-2100, and it is likely that the direct measurement of these events could increase its analytic sensitivity, thus allowing for a direct detection by the instrument to generate an alert signal. Some of the findings for the XE-2100 could potentially be applicable for the Sysmex XS and XT series analysers since they use similar technology [56].

## Future directions

Although current haematology analysers are not specifically designed to detect malaria-related abnormalities, most studies have found sensitivities that comply with WHO malaria-diagnostic guidelines: i.e., ≥95% in samples with >100 parasites/μl [57]. The eventual incorporation of a 'malaria alert' into modern analysers would allow for an automated and adjuvant diagnostic method in the workup of febrile patients possibly infected with malaria, especially in scenarios with low pre-test probability for the disease.

### Detection of unsuspected malaria-infected patients

One of the most important advantages of blood cell analysers is that it would allow for a timely diagnosis of clinically unsuspected malaria cases which otherwise could go undetected leading to adverse clinical outcomes [14]. Initially, a case series by Hänscheid and colleagues reported two 'unsuspected' *P. falciparum *and *Plasmodium ovale *infected patients that were diagnosed by identifying depolarizing events in the side-scatter/depolarized side-scatter plot of a CD 3500 [58]. In another study by Hänscheid and colleagues in Portugal, six patients with clinically unsuspected imported malaria were diagnosed within a 5-month period [39]. For the XE-2100 analyser, Pinter and colleagues in Hungary reported a patient unsuspected of having malaria and later being diagnosed with a *P. vivax *and *Plasmodium malariae *co-infection based on an abnormal DIFF scatter-plot [11]. For the Coulter analysers, Briggs and colleagues found that seven microscopy-negative samples, but positive by fluorescence microscopy and/or immunochromatography, all had a malaria factor ≥3.7 [48].

Consequently, appropriate training of laboratory personnel which validates CBC may allow them to recognize malaria-related changes and request appropriate malaria diagnostic test to confirm the diagnosis. Certainly, automatic flags, either generated by the instrument or the Laboratory Information System would increase the detection of clinical unsuspected cases and would be especially helpful in the developed world where the use of haematology analysers is standard of care, and also in most cities of the developing world where their use is increasingly frequent.

### Automation

Automation by means of a laboratory Information System could be achieved for diagnostic criteria formulated as numerical computations such as the 'malaria factor' obtained for the Coulter GEN**·**S and LH 750 [48,49], and the N-OD1_*Pv *_and N-OD1_*Pf *_models for the Sysmex XE-2100 (Figure 3) [51]. Further development of this diagnostic strategy is needed and would require validation of the models proposed for both Coulter and Sysmex analysers in a wide range of clinical scenarios where other haematologic pathologies may potentially cause false positive results (thrombocytopenia, anaemia or WBC anomalies). The automated computation of multivariate criteria (i.e., considering thrombocytopenia, anaemia and other malaria-related variables) could lead to robust algorithms and a cheap and effective means to screening large number of samples and flag malaria in a case-by-case fashion.

### Improvement of analytic sensitivity and specificity

Since 1980, flow cytometry has been used to detect *Plasmodium *spp. [59] and detection of haemozoin-laden monocytes [29] and the parasite's fluorescence-tagged DNA has been achieved [19,60]. Modern flow cytometry-based haematology analysers could make this technology more accessible for malaria detection. New generation analysers could be built and programmed to specifically detect malaria-related abnormalities and generate accurate malaria-specific alarms. This strategy could improve their analytic sensitivity. For example, one study found that the threshold for malaria detection by a FACStar flow cytometer (Beckton-Dickinson, Mountain View, CA) using fluorescent Hoechst 33258 DNA tagging was ~50 parasites/μl (approximately ~0.001% of infected erythrocytes) [61]. Another study using an experimental Sysmex SIF cytometer (SIF prototype, Sysmex Corporation, Kobe, Japan), a specific cell lysis detergent and nucleic acid fluorescence tagging, was able to detect and differentiate *P. falciparum *parasites by stage with an inferior limit of detection of 0.002% ~ 0.003% infected erythrocytes [60]. This last study hints to the potential of flow cytometry not only to detect a parasitaemia at the range of current expert microscopy, but also to the capacity of these instruments to different parasite forms according to stage of maturity [60].

### Malaria during pregnancy screening using haemozoin-laden monocyte detection

Cell-Dyn detection of depolarizing events could aid in the detection of malaria in pregnant patients. Hänscheid and colleagues assessed the diagnostic accuracy of side-scatter/depolarized side-scatter-plot depolarizing events detected by a CD 3000 to diagnose malaria in pregnant patients (Table 1). This study found that 23.9% (n = 164) were false positives when compared to thick film. However, of these, 37 were further evaluated using PCR, and 14% (n = 5) where found to be true positives [43]. Placental sequestration of parasites decreases the diagnostic yield of microscopy [62]; however, circulating haemozoin-laden macrophages may still be detected by the Cell-Dyn, potentially allowing for detection during prenatal control in malaria endemic regions [43], where CBC counts are available.

### Haemozoin burden and disease severity

Haemozoin load is related to severity and chronicity of malaria, and thus detection and quantification of haemozoin-laden monocytes and granulocytes with the Cell-Dyn instruments could provide a surrogate laboratory marker for disease severity [47,63]. Side-scatter/depolarized side-scatter plot depolarizing events correlate with severe anaemia [42], and one study from Gabon reported more depolarizing green-coded events in children with severe *P. falciparum *malaria [34]. In fact, microscopic determination of these pigment containing cells appears inadequate as severity marker [64], and this may be related to the inherent limitations of microscopic observation, such as the small number of cells that can be observed [65]. Flow cytometry-based cell counts done by haematology analysers could overcome this problem by achieving higher cell counts.

## Conclusions

The early detection of malaria is life-saving. Most health personnel are trained to consider malaria in febrile patients arriving from endemic regions; however, in settings with low pre-test probability for malaria, the diagnosis may be initially overlooked. Malaria detection with haematology analysers, as a by-product of its main purpose, the CBC analysis, can be useful as an adjuvant diagnostic tool in the work-up of febrile patients. Ideally, a flag for malaria could be incorporated and used to guide microscopic evaluation of the patient's blood to establish the diagnosis and start treatment promptly. Automation of a malaria alarm is currently possible for the Coulter GEN·S and LH 750, and for the Sysmex XE-2100 analysers with the help of a Laboratory Information System, though these numerical diagnostic criteria should be validated against population-based samples. Participation of the industry is pivotal for these developments and it would be desirable that haematology analyser manufacturers would be open to evaluate and include algorithms in their instruments that might allow to flag samples with a high suspicion of malaria; a strategy which could potentially assist in generating more accurate algorithms in otherwise simpler devices.

## Abbreviations (alphabetical)

ACT: Artemisinin Combination Therapy; CBC: complete blood count; CD: Cell-Dyn; DNA: deoxyribonucleic acid; FSC: 0° frontal-scatter detection by the Sysmex XE-2100; HIV: Human Immunodeficiency Virus; HRP2: *P. falciparum *histidine-rich protein 2; ICT: immunochromatography; LDH: lactate dehydrogenase; M-OD_*Pv*_: multivariate observer-dependent diagnostic model for *P. vivax *or 'malaria score' which consists of the addition of several variables ('≥7 pixels in the WBC/BASO(III)' [3 points], 'number of granulocyte-coded DIFF abnormalities' [10 variables; 1 point per variable]), with ≥4 points being diagnostic; N-OD: non observer-dependent malaria diagnostic model based on logistic regression where the independent variables are obtained from the data gathered from each blood analysis and the outcome or dependent variable is the predicted probability (PP) for malaria that is dichotomized to positive or negative for malaria based on the optimal PP obtained by ROC analysis; N-OD1: N-OD model that only uses raw numerical variables given by the information processing unit of the Sysmex as independent variables; N-OD1_*Pv *_and N-OD1_*Pf *_are the models for *P. vivax *and *P. falciparum*, respectively; N-OD2: N-OD diagnostic model that uses raw numerical variables given by the information processing unit of the Sysmex as well as pixel counts in the WBC/BASO(III) counting area as independent variables; N-OD2_*Pv *_and N-OD2_*Pf *_are the models for *P. vivax *and *P. falciparum*, respectively; PCR: polymerase chain reaction; PLT-O: optic platelet count; PP: predicted probability for malaria obtained from the logistic regression model; QBC: quantitative buffy coat. RBC: red blood cell(s); RDT: Rapid Diagnostic Tests (for malaria); RDW-SD: The RBC histogram peak height is assumed to be 100%, the distribution width at the 20% frequency level (*y *axis of histogram) is RDW-SD (distance between intersections of histogram and a line extended from the 20% frequency), expressed in femtoliters; ROC: receiver operator curve; RT-PCR: real-time polymerase chain reaction; SD: standard deviation; SFL: 90° side-fluorescence detection by the Sysmex XE-2100; SSC: 90° side-scatter detection by the Sysmex XE-2100; U-OD_*Pv*_: univariate observer-dependent diagnostic model for *P. vivax *which consists of the recognition of a general pattern of abnormalities on several of the Sysmex XE-2100 scatter-plots; VCS: Volume Conductance-Scatter; WBC: white blood cell(s). WHO: World Health Organization; *The following acronyms are product-specific and defined as follows (alphabetical): *ΔDIFF/WBC: Ratio between the WBC count in the DIFF channel over the total WBC count multiplied by 10^3^; DIFF: SSC/SFL WBC separation scatter-plot (Sysmex XE-2100); EOS-I: frontal/depolarized side-scatter WBC separation plot (Cell-Dyn 4000); LYMPH-Y: mean number of events detected in the LYMPH-Y (*y *axis) channel; NEU-EOS: side-scatter/depolarized side-scatter WBC separation plot (Cell-Dyn 4000); RET-EXT: SFL/FSC 'extended reticulocyte' scatter-plot that shows erythrocytes and platelets with varying concentration of nucleic acids (Sysmex XE-2100); WBC/BASO: SSC/FSC WBC separation scatter-plot (Sysmex XE-2100).

## Competing interests

GCZ, TH and MPG: All authors declare that they have no competing financial or any other interest in relation to their work.

## Authors' contributions

GCZ conceived the paper, took the lead in conception and design, and led the drafting of the paper. TH and MPG contributed significantly to the conception and design, and to the writing of the paper. All authors have read and approved the final version of the paper.

## Supplementary Material

Additional file 1 Expanded Table 1 that includes reference diagnostic tests used, blinding status, and observations for each study. Click here for file
