# Supplementary material for: Automated haematology analysis to diagnose malaria
Source: Malar J. 2010 Nov 30;9:346. doi: 10.1186/1475-2875-9-346 (PMC3013084; doi:10.1186/1475-2875-9-346)
Supplement: Additional file 1 — Expanded Table 1 that includes reference diagnostic tests used, blinding status, and observations for each study. [file 1475-2875-9-346-S1.DOC]

Summary of studies evaluating the malaria diagnostic accuracy of Cell-Dyn *series* analysers using the side-scatter/depolarized side-scatter plot abnormal depolarizing events criterion – Including observations for each study and blinding.

| **First author, year and country** | **Number of participants and diagnoses** | **Standard reference test** | **Index test criterion^¶^** | **Blinding** | **Sensitivity %** | **Specificity %** | **Observations** |
| --- | --- | --- | --- | --- | --- | --- | --- |
| Mendelow, 1999, South Africa [18] | Total: 224 directed samples from 175 patients, *P. falciparum:* 93, Species not specified: 2 | Microscopy*, *Pf*HRP2 (Para-sight F) | CD** 3500  ≥1 depolarizing events‡ | + | 72 | 96 | Race differences, probably in relation to immunity. |
| Hänscheid, 2001, Portugal^†^ [39] | Total: 174, *P. falciparum*: 48, *P. vivax*: 6, *P. ovale*: 1, *P. malariae*: 2 | Microscopy | CD 3500  ≥2 depolarizing events | + | 95 | 88 |  |
| Wever, 2002, The Netherlands† [36] | Total: 113, *P. falciparum*: 46, *P. vivax*: 5, *P. ovale*: 4, no differentiation for *P. vivax* or *P. ovale*: 3 | Microscopy, QBC | CD 3500  Either ≥1 depolarizing events or pseudoreticulocytosis | + | 62 | 96 | Depolarizing purple events above a line that starts horizontally through granularity signal channel 25 and then continues at 22.5° angle with the *x* axis. |
| Grobusch, 2003, Germany† [27] | Total: 403, *P. falciparum*: 87, *P. vivax*: 13, *P. ovale*: 5, *P. malariae*: 2 | Microscopy, PCR | CD 3000  ≥1 depolarizing events | + | 48.6 | 96.2 | Semi-immune sensitivity: 73.7%  No- immune sensitivity: 28.6% |
| Scott , 2003, South Africa [35] | Total: 831, *P. falciparum*: 334, *P. vivax*: 7, *P. ovale*: 1, *P. malariae*: 2, mixed or unspecified: 6 | Microscopy, HRP2 (*Pf*/*Pv* ICT), *P*-LDH (OptiMAL), PCR | CD 4000  ≥1 depolarizing events | + | 80.2 | 87.3 | Depolarizing purple events above channel 25 and within the side-scatter/depolarized side-scatter boundary shown in Figure 1 |
| Suh, 2003, South Korea [32] | Total: 168, *P. vivax*: 68 | Microscopy | CD 4000  ≥1 depolarizing events | - | 91.2 | 100 | Considered black or green coded events |
| Dromigny, 2005, Senegal [30] | Total: 799 (directed: suspected of malaria 123, non-suspected random samples 676) *P. falciparum*: 68, treated or subclinical: 83 | QBC, *Pf*HRP2 (MAKROmed), Microscopy, nested PCR | CD 3200  ≥1 depolarizing events | - | Directed  92.9  Random  90.2 | Directed  93.8  Random  96.7 | Depolarizing events above channel 50 and within the side-scatter/depolarized side-scatter boundary shown in Figure 1 |
| Padial, 2005, Equatorial Guinea [41] | Total: 411, *P. falciparum*: 35 , *P. ovale*: 3, mixed: 1 | Microscopy, PCR | CD 4000 | - | 72 | 98 | no exact definition of CD positive criterion given |
| Josephine, 2005, Malaysia [40] | Total: 889, *P. vivax*: 12, *P. malariae*: 3, *P. falciparum*: 1 | Microscopy (peripheral blood smear) | CD 4000 | - | 100 | 100 | no exact definition of CD positive criterion given |
| de Langen, 2006, Namibia [42] | Total: 208, *P. falciparum*: 90 | Microscopy , *Pf*HRP2 (Immuno-Mal) | CD 3700  ≥1 depolarizing events | - | 93 | 97 | no exact definition of CD positive criterion given |
| Hänscheid, 2008, Gabon [34] | Children, total: 368, *P. falciparum*: 152 | Microscopy | CD 3000***  ≥1 depolarizing purple events  Green-coded events | - | a) 96%  b) 85% | a) 96%  b) 96% | Specialized strategy for each type of event*** |
| Hänscheid, 2009, Gabon [43] | Pregnant patients, total 685, *P. falciparum*: 86 | Microscopy | CD 3000  ≥1 depolarizing events | - | 86.8 | 78.5 |  |
| Rathod, 2009, India [33] | Total: 523, *P. falciparum* :73, *P. vivax*: 62 | Microscopy | CD 3200  ≥1 depolarizing events | *-* | *62.2* | *25.3* | *P. vivax* :sensitivity: 63.0%, specificity:61.3% |

**^¶^**Index diagnostic test: abnormal depolarizing events in the side-scatter/depolarized side-scatter plot. ‡All studies use the instrument’s diagonal separation line for eosinophils and neutrophils in the side-scatter/depolarized side-scatter plot , unless otherwise specified. *Microscopy for all studies corresponds to thick film evaluation except where otherwise specified. **CD: Cell-Dyn. IFAT: indirect fluorescence antibody test. QBC: Quantitative buffy coat. PCR: Polymerase chain reaction. ICT: immunochromatography. †Imported malaria. ***For purple events, these were considered positive if present above a line traced at 5 pixels from the *x* axis. For green events, a special gate was created to identify haemozoin-laden granulocytes, with the intention to exclude eosinophils. In accordance with studies using flow cytometric cell sorting [27], the largest possible gate to the left and above the usual location of the eosinophil population was created which did not contain any eosinophils [34]. For this, CBC analyses from children without malaria or pseudoreticulocytosis were used [34].
